# Hsa_circ_0071271 affected the progression of non-small cell lung cancer through miR-23a-5p

**DOI:** 10.1186/s41065-025-00575-5

**Published:** 2025-10-10

**Authors:** Ye Wu, Ling Zhang, Wenhui Li, Dong Yan, Jingjing Yue, Zhusheng Liu

**Affiliations:** 1 Oncology Internal Medicine Ward 4, Guangdong Provincial Agricultural Reclamation Center Hospital, Zhanjiang, 524002 China; 2 Department of Pulmonary and Critical Care Medicine, The People’s Hospital of Dadukou District Chongqing, Chongqing, 400084 China; 3 Internal Medicine Department II, Qingdao Central Hospital, University of Health and Rehabilitation Sciences (Qingdao Central Hospital), Qingdao, 266042 China; 4 Department of Cardiothoracic Surgery, The First People’s Hospital of Zhengzhou, Zhengzhou, 450000 China; 5 Department of Respiratory Medicine, The Affiliated People’s Hospital of Jiangsu University, Zhenjiang, 212002 China; 6 The First People’s Hospital of Zhaoqing City, No. 9, Donggang East Road, Duanzhou District, Zhaoqing, 526060 China

**Keywords:** Lung cancer, Non-small cell lung cancer, Hsa_circ_0071271, MiR-23a-5p, PTEN

## Abstract

**Background:**

Lung cancer ranks among the most prevalent malignancies globally, with non-small cell lung cancer (NSCLC) constituting the predominant subtype. Currently, there are limitations in the treatment options and prognostic evaluation for NSCLC. Hsa_circ_0071271, a non-coding RNA, has an unclear expression and mechanism in NSCLC treatment. In this study, the impacts of hsa_circ_0071271 on NSCLC progression/prognosis and the possible mechanism of its inhibitory role in NSCLC progression through miR-23a-5p were investigated.

**Methods:**

This investigation employed RT-qPCR to initially determine the expression levels of hsa_circ_0071271 in NSCLC tissues and cell lines. To evaluate the clinical significance of hsa_circ_0071271, ROC curve analysis, Kaplan-Meier survival analysis, and Cox regression were conducted. The impact of hsa_circ_0071271 knockdown on NSCLC cell lines A549 and CALU3 was examined through CCK-8 assays, flow cytometry, and transwell assays, corresponding to cell proliferation, apoptosis, and migration/invasion. The dual-luciferase reporter assay was used to examine the relationships between miR-23a-5p and hsa_circ_0071271, as well as between PTEN and miR-23a-5p. Pearson correlation analysis was conducted to assess the correlation between PTEN and miR-23a-5p. Subsequent experiments with CCK-8, flow cytometry, and transwell assays were carried out to explore how hsa_circ_0071271 regulates miR-23a-5p/PTEN and thereby affects NSCLC cell proliferation, apoptosis, migration, and invasion.

**Results:**

Hsa_circ_0071271 was expressed highly in NSCLC tissues and multiple cell lines. Hsa_circ_0071271 effectively distinguishes tumor tissues from normal ones and is associated with patient survival rates. Knocking down hsa_circ_0071271 inhibits NSCLC cell proliferation and migration/invasion while promoting apoptosis. The study also revealed an interaction between hsa_circ_0071271 and miR-23a-5p, as well as between PTEN and miR-23a-5p, with their expression levels showing a significant negative correlation. Further experiments indicated that hsa_circ_0071271 regulates miR-23a-5p/PTEN to suppress NSCLC cell proliferation, migration, and invasion and promote apoptosis.

**Conclusions:**

Regulating miR-23a-5p/PTEN by hsa_circ_0071271 knockdown has been found to inhibit NSCLC cell proliferation, migration and invasion, as well as promote apoptosis.

## Background

Globally, lung cancer stands out as one of the most frequently occurring cancers and serves as the primary contributor to cancer-related mortality. Annually, around 2.2 million individuals are newly diagnosed with lung cancer [1]. Projections indicate that by 2035, the annual number of lung cancer-associated fatalities may climb to 3 million [2]. From a clinical perspective, lung cancer is classified into non-small cell lung cancer (NSCLC) and small cell lung cancer. NSCLC represents the predominant category, constituting approximately 85% of all lung cancer cases. Within NSCLC, the primary subtypes are squamous cell carcinoma (SCC), adenocarcinoma (ADC), and large cell carcinoma (LCC) [3]. Tumors of this kind are notorious for their exceptionally high recurrence rate, which varies between 35% and 50%, thus posing a significant therapeutic challenge [4]. The International Association for the Study of Lung Cancer (IASLC) has established a staging system for lung cancer that takes into account tumor size, lymph node involvement, and the presence of distant metastases. According to this system, lung cancer is categorized into four stages, from I to IV. Lung cancers in stages I and II are considered early-stage, whereas those in stages III and IV are classified as advanced [5].

Currently, in NSCLC diagnosis, conventional tools like chest X-rays and sputum cytology, with insufficient sensitivity for early-stage detection, are widely used. Moreover, conventional tumor markers are characterized by low sensitivity and specificity, which pose significant challenges for the early diagnosis of lung cancer [2]. Despite progress in lung cancer research and treatment, and the use of various therapies (surgery, radiotherapy, chemotherapy, molecular-targeted therapy, immunosuppressants), the overall survival rate remains low [6, 7]. To improve survival, early diagnosis and precise treatment are key [8]. Thus, more specific and non-invasive biomarkers are needed in the detection and staging of lung cancer.

Non-coding RNAs (ncRNAs), which are RNA molecules not translated into proteins, are key regulators of gene expression and are implicated in cancer development as oncogenes or tumor suppressors [6]. Circular RNAs (circRNAs), a type of ncRNAs, are conserved and stable endogenous RNAs that contribute to gene regulation. They form a covalently closed ring through back-splicing [9], which protects them from nucleases. This makes them potential biomarkers for cancer diagnosis and prognosis [10]. Studies show that circRNAs drive tumor progression by regulating cell proliferation, metastasis, and apoptosis [11]. For instance, in lung adenocarcinoma (LUAD), circRNA expression is significantly linked to cancer development [6], circPRKCI promotes LUAD by upregulating E2F7 via competing for miR-545/miR-589 binding [12], circNDUFB2 has an inhibitory effect on NSCLC development by destabilizing IGF2BPs as well as enhancing anti - tumor immune responses [13].

Circular RNAs (circRNAs) can function as miRNA sponges by containing miRNA response elements (MREs), thereby inhibiting miRNA activity. MiRNAs typically bind to the 3’ untranslated regions (3’UTRs) of mRNAs, resulting in translation suppression or mRNA degradation [11], and are closely linked to NSCLC. For example, miR-506 is upregulated in 83% of NSCLC patients and correlated with reactive oxygen species (ROS) levels [14]. And downregulated miR-487a-3p promotes NSCLC progression by targeting Smad7 [6].

This study explored how hsa_circ_0071271 affects NSCLC progression and revealed its mechanism of inhibiting NSCLC via miR-23a-5p, offering potential targets for NSCLC diagnosis and therapy.

## Materials and methods

### Human tissue specimens

Tumor and normal tissue specimens were resected surgically from 127 lung cancer patients who had not undergone preoperative radiation, chemotherapy, or targeted therapy. Written informed consent was obtained from all patients, and the study protocol was approved by The First People’s Hospital of Zhengzhou Ethics Committee.

### Cell culture

The study utilized six cell lines (Feng Hui Biotechnology): one normal human bronchial epithelial cell line (16HBE) and five NSCLC cell lines, namely A549, NCI-H292, NCI-H596, NCI-H1299, and CALU-3. These cells were grown in RPMI-1640 medium (Hyclone) supplemented with 10% fetal bovine serum (Gibco, 10270106).

### Si-circ, pcDNA-circ, miRNA mimic, miRNA inhibitor, oe-NC, OE-PTEN and transfection experiments

The si-circ and pcDNA-circ targeting hsa_circ_0071271, the miR-23a-5p mimic and inhibitor, as well as the oe-NC and OE-PTEN plasmids, were acquired from GenePharma (China), along with non-targeting negative controls. Transfection experiments were conducted using Lipofectamine 2000 (Invitrogen) to introduce these constructs into the cells.

### Cell counting kit-8 (CCK-8) assay

Cellular viability was assessed using the CCK8 assay kit (Beyotime Biotechnology, C0038). Cells were seeded in a 96-well plate at 100 µL per well and subjected to various treatments as per the experimental design. Cell viability was quantified with a microplate reader (Thermo Fisher Scientific) by measuring absorbance at 450 nm.

### Cell apoptosis by flow cytometry

Cell resuspension in binding buffer containing FITC-conjugated Annexin V and 1 mL of PI (Invitrogen) was used to detect cell apoptosis. Flow cytometry (BD Biosciences) was used to analyze apoptotic cells.

### Dual luciferase reporter gene experiment

First, the GR 3’-UTR fragment into the pmirGLO dual-luciferase reporter vector (Promega, Madison, WI, USA) to construct wild-type (circ-WT) and mutant (circ-MUT) reporter plasmids. Prepare 16HBE cells. 16HBE cells were co-transfected with these vectors and miR-23a-5p mimics (or mimic control) / inhibitors (or inhibitor control)using Lipofectamine 3000 (Thermo Fisher Scientific, L3000015). At the 48-hour mark, the Dual-Luciferase Reporter Assay System (Promega Corporation, E1910) was employed to measure luciferase activity, with Firefly Luciferase measured first, followed by Renilla Luciferase.

### Transwell assay

Cell migration and invasion capabilities were evaluated using Transwell chambers (8.0 μm pore size; Corning, 3422) in 24-well plates. For invasion assays, chambers were coated with Matrigel (BD Biosciences, 354230). Cell suspensions were seeded in the upper chamber, while the lower chamber contained RPMI-1640 medium supplemented with 10% FBS. Uncoated chambers served for migration assays. Cells that migrated and invaded were fixed, stained, and photographed under an optical microscope (PRECISE), and the number of cells that migrated and invaded into the lower chamber was recorded.

### Reverse transcription-quantitative PCR (RT-qPCR)

Total RNA extraction from lung cancer tissues and cell lines was performed using the RNA/Protein Isolation Kit (Beyotime Biotechnology, R0018S) per the manufacturer’s protocol. Reverse transcription was carried out with the PrimeScript™ RT reagent Kit (Takara, RR037Q). For qPCR, 1 µL of the resulting cDNA was amplified using Power SYBR™ Green PCR Master Mix (Thermo Fisher Scientific, 4367659) on a QuantStudio Dx Real-Time PCR System (Thermo Fisher Scientific). β-actin and U6 served as internal reference standards.

### Statistical analysis

Data are presented as mean ± SD from at least three independent experiments with repeated measurements. Statistical analysis was conducted using GraphPad Prism 9.3.1. The independent samples t-test compared two groups, while one-way ANOVA was used for multiple groups. ROC curves were generated in MedCalc, and Kaplan-Meier survival curves along with Cox regression analyses were performed in IBM SPSS Statistics 23. A *p*-value threshold of 0.05 was set for statistical significance.

## Results

### Hsa_circ_0071271 is up-regulated in NSCLC tissues and cell lines

**Fig. 1:**
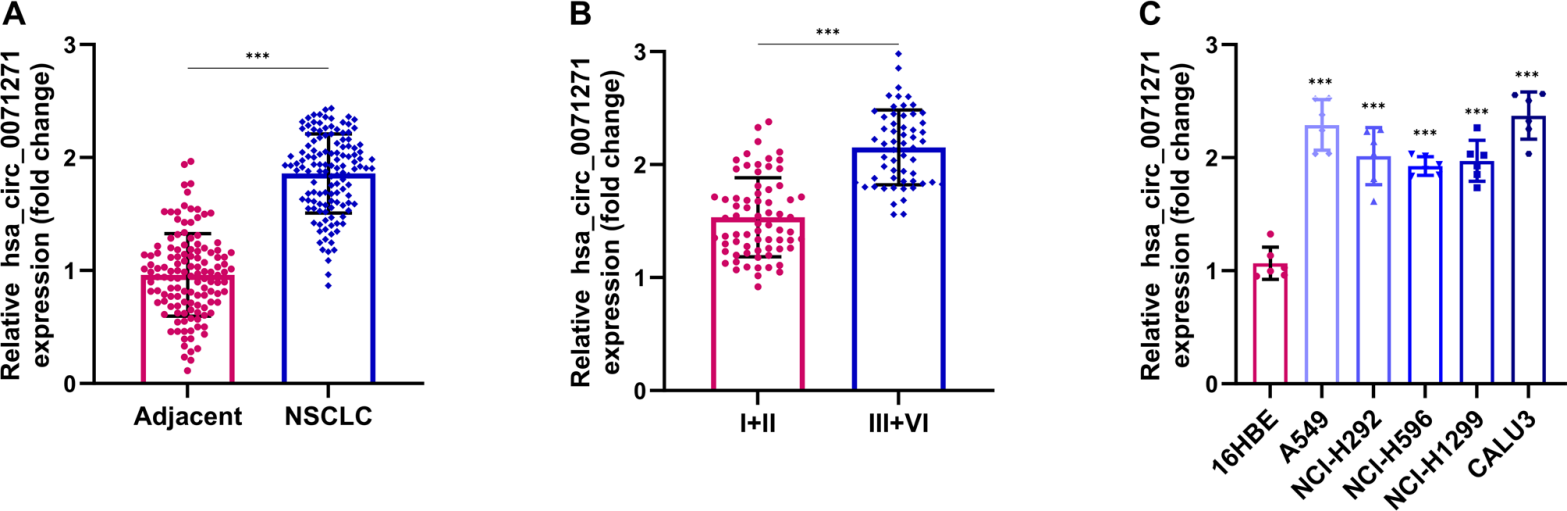
Expression levels of hsa_circ_0071271 in NSCLC tissues and cell lines. (**A**) Expression levels of hsa_circ_0071271 in adjacent non-tumor and NSCLC tumor tissues. (**B**) Expression levels of hsa_circ_0071271 in NSCLC tumor tissues of different stages. (C) Expression levels of hsa_circ_0071271 in normal cells (16HBE) and NSCLC cell lines (A549, NCI-H292, NCI-H596, NCI-H1299, CALU-3). ***p < 0.001

In this study, RT-qPCR was first used to detect hsa_circ_0071271 expression in NSCLC tissues and cell lines. The results revealed that hsa_circ_0071271 was significantly up-regulated in NSCLC tumor tissues compared to para-tumorous tissues (Fig. 1A). Further analysis of hsa_circ_0071271 expression across different NSCLC stages showed a significant increase in late-stage tumor tissues compared to early-stage ones (Fig. 1B). Additionally, hsa_circ_0071271 expression was compared between NSCLC cell lines (A549, NCI-H292, NCI-H596, NCI-H1299, CALU-3) and 16HBE cells. The results indicated that hsa_circ_0071271 expression was significantly higher in NSCLC cells than in 16HBE cells (Fig. 1C).

### Hsa_circ_0071271 shows potential for NSCLC diagnosis and prognosis

**Fig. 2:**
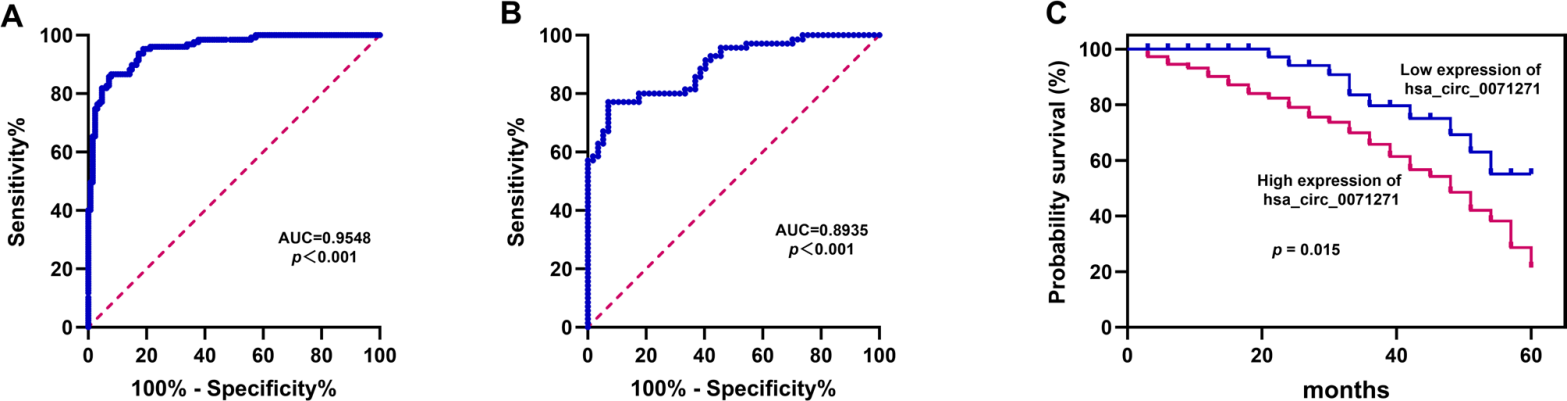
The diagnostic and prognostic potential of hsa_circ_0071271 in NSCLC. (**A**) ROC curve of hsa_circ_0071271 (Adjacent vs. NSCLC, Sensitivity = 92.9%, Specificity = 85.8%). (**B**) ROC curve of hsa_circ_0071271 (NSCLC I + II vs. NSCLC III + IV, Sensitivity = 93%, Specificity = 77.1%). (**C**) Kaplan-Meier survival curve of hsa_circ_0071271 (NSCLC tumor tissues were grouped by the average hsa_circ_0071271 mRNA expression level; High expression of hsa_circ_0071271 (*n* = 74); Low expression of hsa_circ_0071271 (*n* = 53)

Based on 127 NSCLC patients’ data (Table 1), ROC curve analysis, Kaplan-Meier survival analysis, and Cox regression were applied to evaluate hsa_circ_0071271’s potential in NSCLC diagnosis and prognosis. Notably, the ROC curve analysis of hsa_circ_0071271 between NSCLC tumor tissues and adjacent non-tumor tissues, as well as between early-and late-stage NSCLC tumor tissues, showed a remarkable area under the curve (AUC) of 0.9548 (*p* < 0.001), indicating its high diagnostic efficacy for distinguishing tumor tissues from adjacent non-tumor tissues (Fig. 2A), indicating diagnostic potential. For differentiating early-from late-stage NSCLC, the AUC was 0.8935 (*p* < 0.001) (Fig. 2B), confirming its ability to distinguish tumor stages. Kaplan-Meier analysis, based on hsa_circ_0071271 mRNA expression levels in NSCLC tumor tissues, showed that patients exhibiting elevated hsa_circ_0071271 levels were associated with significantly lower survival rates when compared to those with lower expression levels. (*p* = 0.015) (Fig. 2C), indicating its potential as a prognostic indicator. Cox regression analysis further identified hsa_circ_0071271 as the sole factor among those analyzed with *p* < 0.05 (*p* = 0.022), underscoring its significance in prognosis (Table 2).

**Table 1:** Subjects clinicopathological parameter

Clinicopathological parameter	Number of patients	hsa_circ_0071271		*p*
		Low expression (*n* = 53)	High expression (*n* = 74)	
Gender				
Male	62	22	40	0.163
Female	65	31	34	
Age				
>60	79	36	43	0.261
≤ 60	48	17	31	
BMI (kg/m^2^)				
>23.9	64	27	37	0.916
≤ 23.9	63	26	37	
Smoking				
Yes	66	24	42	0.202
No	61	29	32	
Tumor size (cm)				
>7	65	21	44	0.027
≤ 7	62	32	30	
Distant metastasis				
Yes	64	20	44	0.016
No	63	33	30	
CEA (ng/mL)				
>5	66	21	45	0.018
≤ 5	61	32	29	

**Table 2:** A multivariate Cox regression analysis was conducted to assess the prognostic significance of hsa_circ_0071271

Parameters	HR factor	95% CI	*p*
hsa_circ_0071271	2.580	1.143–5.882	0.022
Gender	1.157	0.604–2.217	0.661
Age	1.447	0.754–2.776	0.267
BMI (kg/m^2^)	1.296	0.692–2.427	0.419
Smoking	1.274	0.680–2.385	0.450
Tumor size (cm)	1.259	0.462–3.428	0.652
Distant metastasis	1.241	0.451–3.416	0.676
CEA (ng/mL)	1.265	0.657–2.435	0.482

### Knockdown of hsa_circ_0071271 expression inhibits NSCLC tumor cell progression

**Fig. 3:**
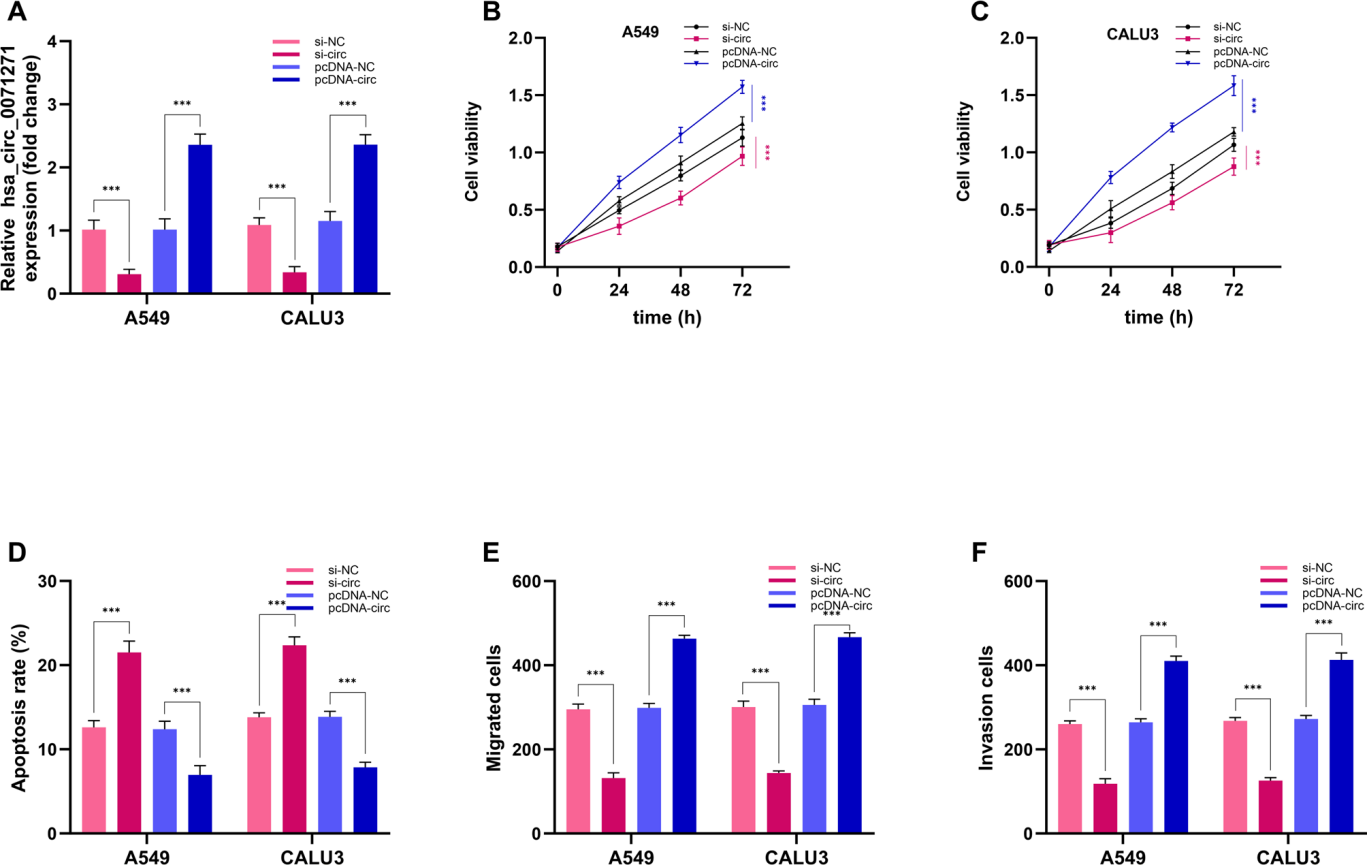
Effect of hsa_circ_0071271 on proliferation, apoptosis, migration, and invasion of A549 and CALU3 cells. (**A**) Hsa_circ_0071271 expression in A549 and CALU3 cells. (**B**) A549 cell proliferation. (**C**) CALU3 cell proliferation. (**D**) Apoptosis in A549 and CALU3 cells. (**E**) Migration in A549 and CALU3 cells. (F) Invasion in A549 and CALU3 cells. ***p < 0.001

The impact of hsa_circ_0071271 on NSCLC cells (A549, CALU3) was examined in this study, showing significant impacts on their proliferation, apoptosis, migration, and invasion. Transfection experiments revealed that in the si-circ group, in A549 and CALU3 cells, hsa_circ_0071271 expression was markedly diminished compared to the si-NC and pcDNA-NC groups, while the pcDNA-circ group showed significantly increased expression (Fig. 3A), indicating successful knockdown and overexpression of hsa_circ_0071271. In terms of proliferation, the si-circ group had lower cell viability than the si-NC group at all time points, whereas the pcDNA-circ group had higher viability than the pcDNA-NC group (Fig. 3B-C). This suggests hsa_circ_0071271 knockdown suppresses proliferation, while its overexpression promotes it. Regarding apoptosis, the si-circ group had a higher apoptosis rate than the si-NC group, and the pcDNA-circ group had a lower rate than the pcDNA-NC group (Fig. 3D), indicating that hsa_circ_0071271 knockdown promotes apoptosis, while overexpression inhibits it. In migration and invasion assays, the si-circ group exhibited fewer migrated and invaded cells than the si-NC group, whereas the pcDNA-circ group showed an increase in migrated and invaded cells compared to the pcDNA-NC group. (Fig. 3E-F), implying hsa_circ_0071271 knockdown inhibits these processes, while overexpression promotes them. Overall, the suppression of hsa_circ_0071271 could inhibit cell proliferation, migration, invasion and induce apoptosis.

### Hsa_circ_0071271 interacts with miR-23a-5p

**Fig. 4:**
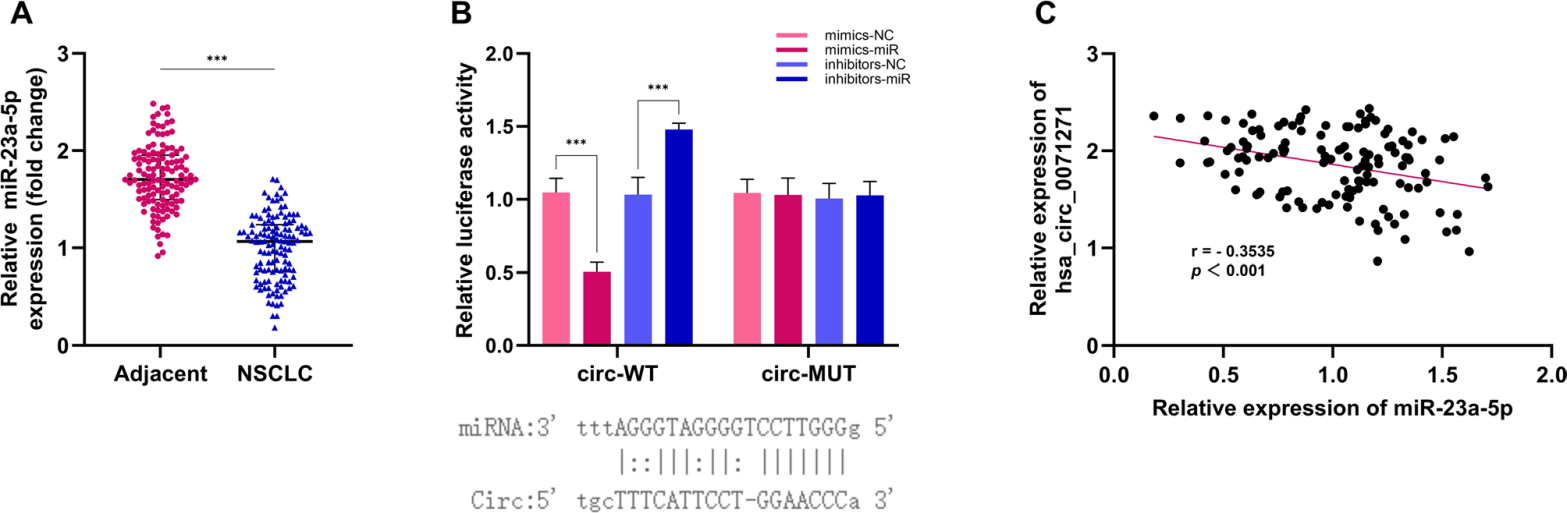
MiR-23a-5p expression and its interaction with hsa_circ_0071271. (**A**) Expression of miR-23a-5p in adjacent non-tumor and NSCLC tumor tissues. (**B**) Interaction between hsa_circ_0071271 and miR-23a-5p. (**C**) The relationship between hsa_circ_0071271 and miR-23a-5p. ***p < 0.001

This study first detected miR-23a-5p expression in NSCLC tissues via RT-qPCR and found it was downregulated compared to adjacent tissues (Fig. 4A). The binding site prediction results between hsa_circ_0071271 and miR-23a-5p were obtained from the circBank database (www.circbank.cn/). The dual-luciferase reporter assay results indicated that in comparison to the mimics-NC group, the circ-WT luciferase activity was significantly reduced in the mimics-miR group, indicating miR-23a-5p can bind to hsa_circ_0071271 and inhibit its luciferase activity. The luciferase activity of circ-MUT showed no significant difference between the mimics-miR and mimics-NC groups, showing miR-23a-5p doesn’t bind to the mutated site. Additionally, compared to the inhibitors-NC group, the luciferase activity of circ-WT was significantly increased in the inhibitors-miR group. In contrast, no significant change in circ-MUT’s luciferase activity was observed between the inhibitors-miR and inhibitors-NC groups (Fig. 4B). These results confirm the specific binding of miR-23a-5p to hsa_circ_0071271. Furthermore, scatter plot analysis demonstrated a significant negative correlation between hsa_circ_0071271 and miR-23a-5p expression levels in NSCLC tissues. (*r*∉=∉-0.3535) (Fig. 4C).

### Hsa_circ_0071271 affects NSCLC tumor progression by regulating miR-23a-5p

**Fig. 5:**
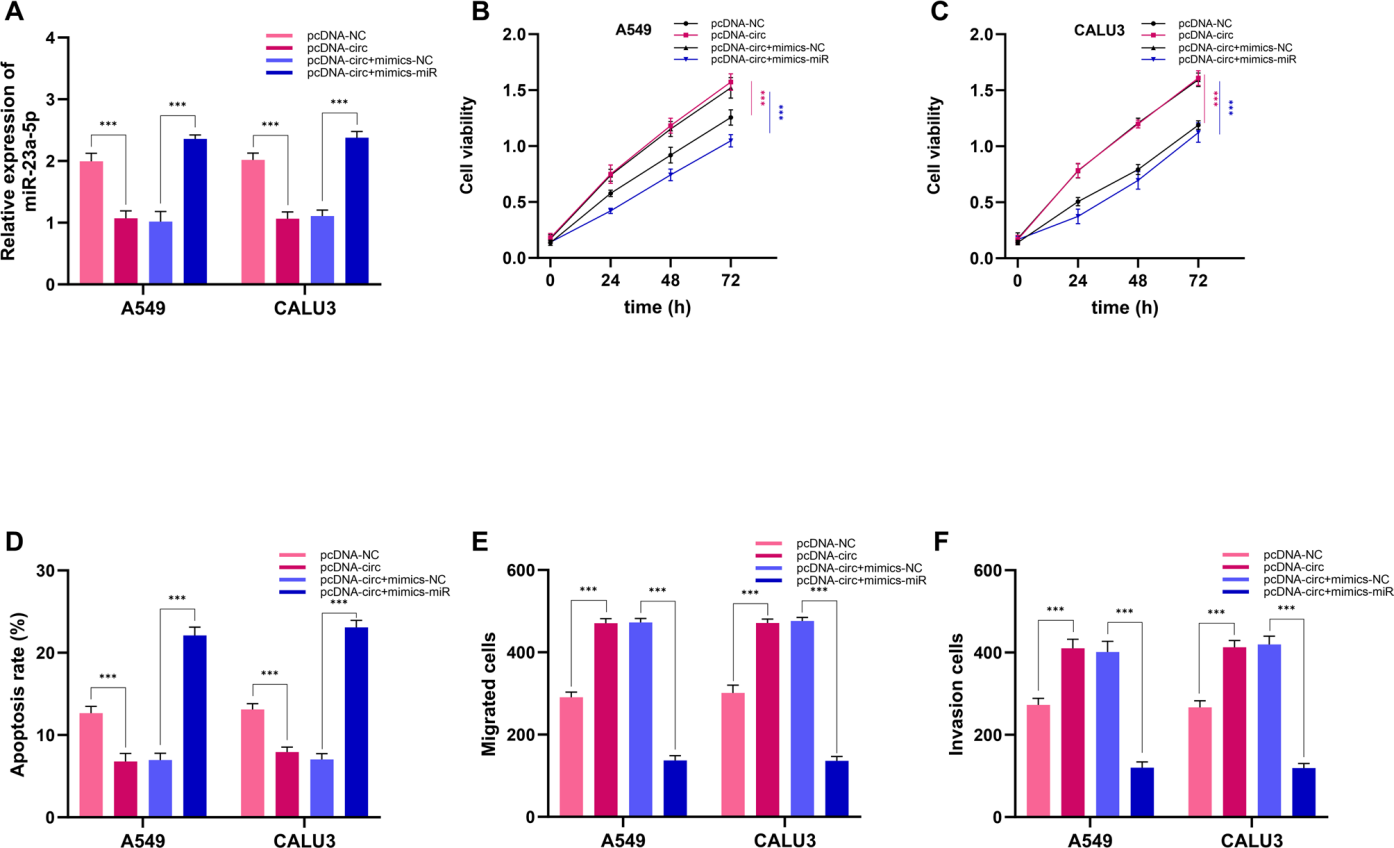
Effect of hsa_circ_0071271 on A549 and CALU3 cells via miR-23a-5p regulation. (**A**) Expression levels of miR-23a-5p mRNA in A549 and CALU3 cells. (**B**) Proliferation of A549 cells. (**C**) Proliferation of CALU3 cells. (**D**) Apoptosis in A549 and CALU3 cells. (**E**) Migration of A549 and CALU3 cells. (**F**) Invasion of A549 and CALU3 cells. ***p < 0.001

Transfection assays indicated that in A549 and CALU3 cells, miR-23a-5p expression was lower in the pcDNA-circ group than in the pcDNA-NC group. Co-transfection with mimics-miR significantly increased miR-23a-5p expression (Fig. 5A), confirming successful transfection. Regarding cell proliferation, the pcDNA-circ group showed higher cell viability than the pcDNA-NC group. Co-transfection with mimics-miR reduced cell viability (Fig. 5B-C), indicating that mimics-miR can reverse the proliferation-promoting effect of pcDNA-circ. For apoptosis, the pcDNA-circ group had a lower apoptosis rate than the pcDNA-NC group. Co-transfection with mimics-miR increased the apoptosis rate (Fig. 5D), demonstrating that mimics-miR can counteract the apoptosis-inhibiting effect of pcDNA-circ. In terms of cell migration and invasion, the pcDNA-circ group had more migrated and invaded cells than the pcDNA-NC group. Co-transfection with mimics-miR reduced the number of migrated and invaded cells (Fig. 5E-F), proving that mimics-miR can nullify pcDNA-circ’s enhancing effect on migration and invasion.

### PTEN interacts with miR-23a-5p

**Fig. 6:**
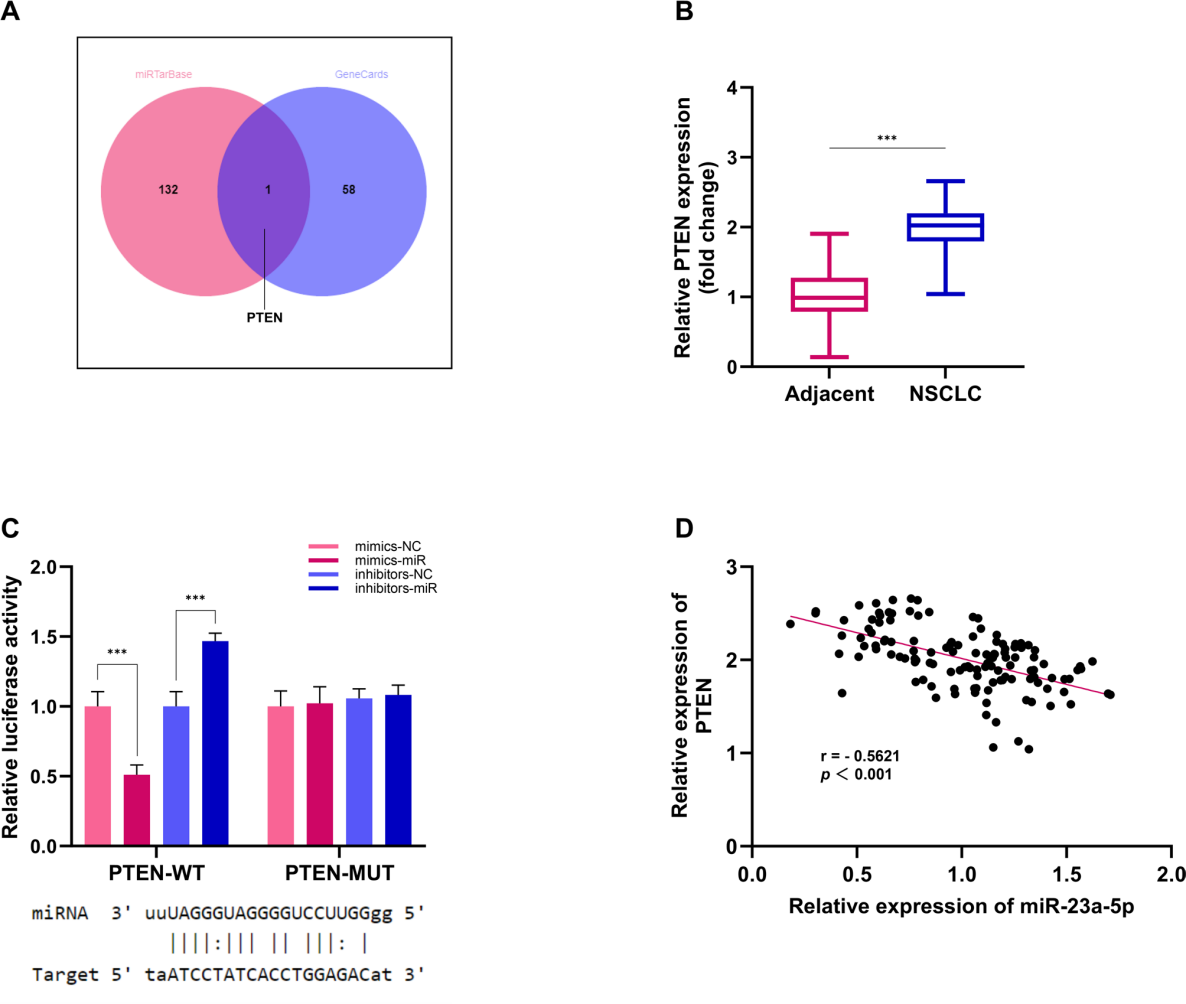
The interaction between PTEN and miR-23a-5p. (**A**) The identification of downstream targets of miR-23a-5p using a Venn diagram. (**B**) The expression levels of PTEN in adjacent normal tissues and NSCLC tumor tissues. (**C**) The interaction between PTEN and miR-23a-5p. (**D**) The correlation between PTEN and miR-23a-5p. ***p < 0.001

We first predicted miR-23a-5p’s downstream genes via the miRDB database (https://mirdb.org/) and identified NSCLC-related genes on GeneCards (www.genecards.org). The intersection of these two datasets revealed PTEN as a common target (Fig. 6A). We then measured PTEN expression in NSCLC tissues and found it significantly upregulated in tumor tissues compared to adjacent normal tissues (Fig. 6B), indicating PTEN overexpression in NSCLC. Next, using the miRTarBase database (https://miRTarBase.cuhk.edu.cn/), we predicted the binding sites between PTEN and miR-23a-5p and validated their interaction through a dual-luciferase reporter assay. The PTEN wild-type (PTEN-WT) and mutant (PTEN-MUT) sequences were cloned into the pmirGLO vector and co-transfected with miR-23a-5p mimics or a negative control into cells. The results showed that miR-23a-5p significantly inhibited luciferase activity of the PTEN-WT vector but not the PTEN-MUT vector, while the miR-23a-5p inhibitor enhanced PTEN-WT luciferase activity with no effect on PTEN-MUT (Fig. 6C). Additionally, Pearson correlation analysis revealed a significant negative correlation between PTEN and miR-23a-5p (*r*∉=∉-0.5621, *p* < 0.001, Fig. 6D).

### Hsa_circ_0071271 affects NSCLC tumor progression through miR-23a-5p/PTEN

**Fig. 7:**
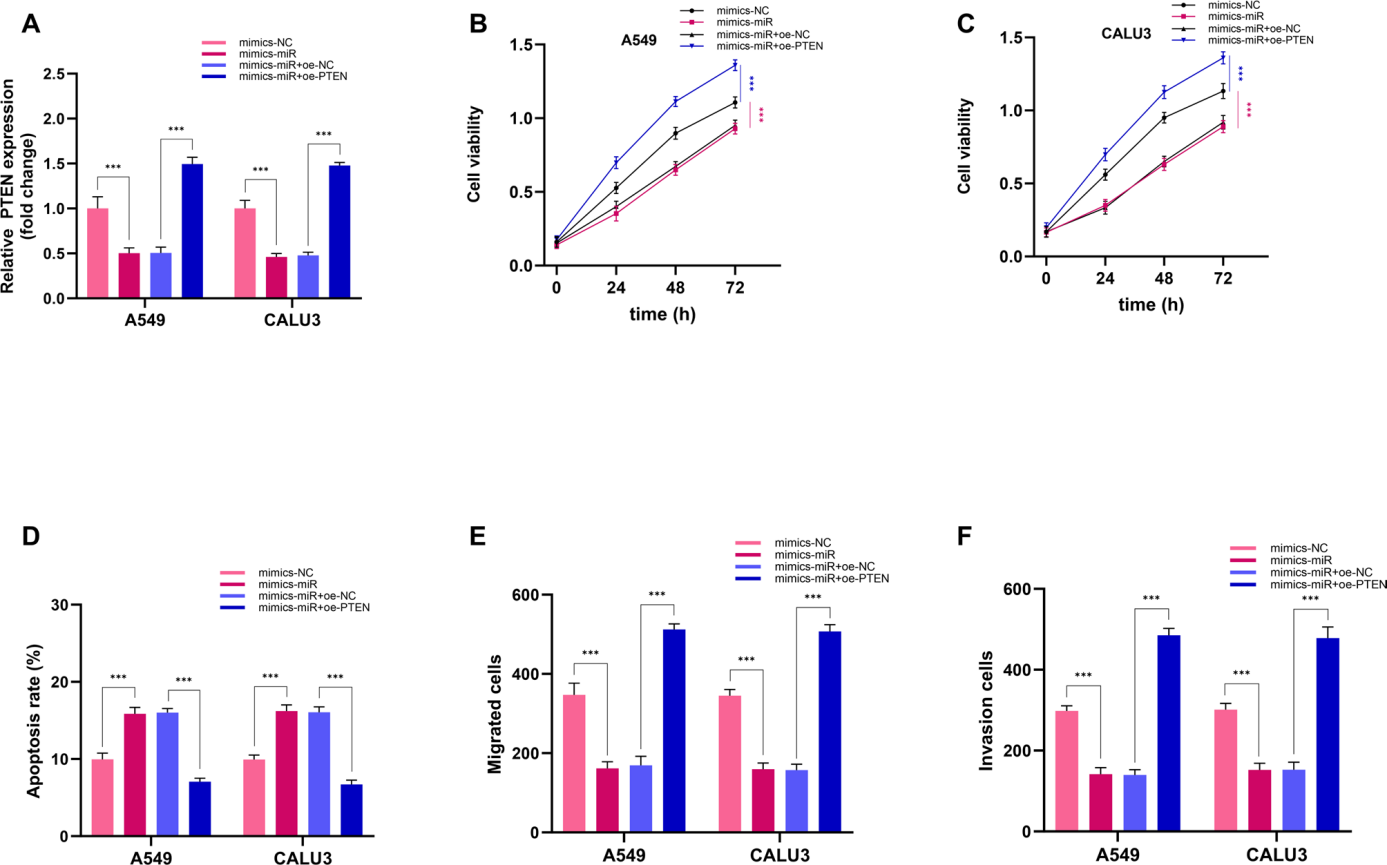
The influence of hsa_circ_0071271 on the progression of NSCLC through miR-23a-5p/PTEN. (**A**) PTEN mRNA expression levels in different groups. (**B**) A549 cell viability in different groups. (**C**) CALU3 cell viability in different groups. (**D**) Cell apoptosis rate in different groups. (**E**) Cell migration number in different groups. (**F**) Cell invasion number in different groups. ***p < 0.001

In this study, transfection experiments in A549 and CALU3 cells demonstrated that PTEN expression was markedly reduced in the mimics-miR group compared to the mimics-NC group, while PTEN expression was significantly increased in the mimics-miR + oe-PTEN group compared to the mimics-miR + oe-NC group (Fig. 7A), confirming the successful transfection of miR-23a-5p and oe-PTEN. Subsequent analysis of cell proliferation revealed that the mimics-miR group exhibited significantly decreased cell viability, an effect that was reversed by PTEN overexpression (mimics-miR + oe-PTEN group) (Fig. 7B-C). In terms of apoptosis, the mimics-miR group showed a significantly higher apoptosis rate than the control group, and this increase was abrogated by PTEN overexpression (Fig. 7D). Furthermore, in migration and invasion assays, the number of migrated and invaded cells was significantly lower in the mimics-miR group than in the mimics-NC group. However, co-transfection with oe-PTEN notably increased the number of migrated and invaded cells, indicating that PTEN overexpression could reverse the inhibitory effects of miR-23a-5p on the migration and invasion of A549 and CALU3 cells (Fig. 7E-F). Collectively, these findings indicate that PTEN overexpression can effectively counteract the effects of miR-23a-5p on the proliferation, apoptosis, migration, and invasion of A549 and CALU3 cells.

## Discussion

The incidence of lung cancer is rising, primarily due to carcinogens in tobacco smoke [2]. Currently, lung cancer diagnosis predominantly depends on CT and tissue biopsy. However, CT has limitations in diagnostic accuracy and tissue biopsy is an invasive method. Consequently, there is a need to enhance the sensitivity and specificity of existing non-invasive biomarkers [15]. Additionally, factors like tumor location, pathological type, and metastasis complicate diagnosis, causing over half of patients to be diagnosed with metastatic tumors [16]. NSCLC is the most common type, often linked to poor outcomes due to late diagnosis and limited treatment options [17]. Research has indicated that early diagnosis is closely tied to improved survival rates [5], so accurate early diagnosis and timely treatment are crucial for reducing lung cancer mortality.

MiRNAs and other ncRNAs form regulatory networks by targeting multiple gene mRNAs. Other ncRNAs (circRNAs) can interact with miRNAs to affect their stability and play a key regulatory role in cellular functions. This interplay is widely impactful and common in cancers [18]. For instance, in esophageal cancer, circBCAR3 accelerates tumorigenesis by binding to miR-27a-3p and upregulating TNPO1 [19]. Studies indicate a close link between ncRNAs and lung cancer development [18]. For instance, exosome-delivered circVMP1 promotes NSCLC progression via the miR-524-5p axis [11]. Conversely, CircVAPA drives SCLC progression via the miR-377-3p and miR-494-3p axis [20]. Thus, identifying specific and cell-friendly biomarkers could improve NSCLC diagnosis and prognosis.

This study delves into hsa_circ_0071271 expression in NSCLC and its mechanism of inhibiting NSCLC progression through miR-23a-5p/PTEN, while evaluating its potential diagnostic and prognostic significance in NSCLC. Research indicates that hsa_circ_0071271 is overexpressed in NSCLC tissues and cell lines, with higher expression in late-stage tumors. ROC curve analysis shows that hsa_circ_0071271 can distinguish tumor tissues from adjacent and early-stage from late-stage NSCLC tissues, suggesting diagnostic potential. Kaplan-Meier analysis reveals a higher survival rate in the low-expression group, and Cox regression shows that hsa_circ_0071271 has significant prognostic value. Knocking it down can inhibit NSCLC cell proliferation, migration, and invasion and promote apoptosis. Additionally, miR-23a-5p is downregulated in NSCLC tissues, with a negative correlation between its expression and that of hsa_circ_0071271. Dual-luciferase reporter assays confirm their interaction. Further experiments indicate that miR-23a-5p expression in NSCLC tissues is significantly lower than in adjacent normal tissues, while PTEN is overexpressed. Dual-luciferase reporter assays confirmed significant negative correlations between hsa_circ_0071271 and miR-23a-5p, and between miR-23a-5p and PTEN. Further experiments demonstrated that hsa_circ_0071271 influences NSCLC progression by regulating the miR-23a-5p/PTEN axis, significantly affecting the proliferation, apoptosis, migration, and invasion of A549 and CALU3 cells. Overall, hsa_circ_0071271 acts as a suppressor of NSCLC progression via the miR-23a-5p/PTEN pathway. However, more evidence is needed to confirm its efficacy and safety before it can serve as a potential therapeutic target for NSCLC.

## Conclusion

To sum up, hsa_circ_0071271 is overexpressed in NSCLC tissues and diverse NSCLC cell lines. Silencing hsa_circ_0071271 exerts an inhibitory effect on NSCLC cell proliferation, migration, and invasion, and enhances apoptosis. It likely hinders NSCLC progression via miR-23a-5p /PTEN regulation. Hsa_circ_0071271 shows promise for NSCLC diagnosis and prognosis.

## Data Availability

Corresponding authors may provide data and materials.

## Abbreviations

ADC: Adenocarcinoma
circRNAs: Circular RNAs
NSCLC: Non-small cell lung cancer
SCC: Squamous cell carcinoma
LCC: Large cell carcinoma
IASLC: International Association for the Study of Lung Cancer
ncRNAs: Non-coding RNAs
LUAD: Lung adenocarcinoma
MREs: miRNA response elements
